# Long-term efficacy and safety of anti-VEGF therapy in retinitis pigmentosa: a case report

**DOI:** 10.1186/s12886-018-0914-z

**Published:** 2018-09-14

**Authors:** Manabu Miyata, Akio Oishi, Maho Oishi, Tomoko Hasegawa, Hanako Ohashi Ikeda, Akitaka Tsujikawa

**Affiliations:** Department of Ophthalmology and Visual Sciences, Kyoto University Graduate School of Medicine, Shogoin Kawahara-cho 54, Sakyo-ku, Kyoto, 606-8507 Japan

**Keywords:** Retinitis pigmentosa, Anti-VEGF therapy, Choroidal neovascularization, Long-term, PRPH2

## Abstract

**Background:**

Retinitis pigmentosa (RP), a neurodegenerative disease, is occasionally accompanied by choroidal neovascularization (CNV) and cystoid macular oedema. It is presently treated with repeated intravitreal injections of anti-vascular endothelial growth factor (VEGF) agents. However, there are concerns regarding long-term inhibition of VEGF by the use of these agents, especially in cases involving neurodegenerative diseases, since VEGFs have a neuroprotective effect. Currently, there are no reports on the long-term safety of anti-VEGF therapy in patients with RP.

**Case presentation:**

In this report, we describe the case of a 56-year-old female patient with CNV associated with RP who was treated with anti-VEGF therapy for 8 years. She had autosomal dominant RP with a heterozygous *PRPH2* mutation (c.410G > A) and complained of metamorphopsia in her left eye. Examinations revealed CNV with serous retinal detachment. She was treated with as-needed injections for 2 years; however, she experienced a recurrence. Therefore, we switched to a bimonthly regimen that was continued for 6 years. In total, the patient received 34 injections of various types of anti-VEGFs over 8 years. No recurrences were noted during that time, and we have not detected any negative effects concerning the progression of visual field loss in comparison with the fellow eye.

**Conclusions:**

No negative effects related to the progression of visual field loss were observed during continuous treatment with anti-VEGF agents for 8 years in our patient.

## Background

Retinitis pigmentosa (RP) causes progressive vision loss due to the degeneration of rod and cone photoreceptors [1]. The disease is occasionally accompanied by choroidal neovascularization (CNV) [2], which is presently treated with repeated intravitreal injections of anti-vascular endothelial growth factor (VEGF) agents. However, VEGFs have a neuroprotective effect [3]; for example, loss of VEGF-A from the retinal pigment epithelium damages the choriocapillaris, which leads to photoreceptor dysfunction [4]. Therefore, there are concerns regarding long-term inhibition of VEGF, particularly in patients with neurodegenerative diseases such as RP. If not for these concerns, the frequency of use of anti-VEGF therapy for cystoid macular oedema (CME) secondary to RP would increase because of its effectiveness [5, 6]. However, there are no reports on the long-term efficacy and safety of anti-VEGF therapy for patients with RP. Herein, we present the case of a patient who had CNV associated with RP that was treated with anti-VEGF for 8 years.

## Case presentation

**Fig. 1:**
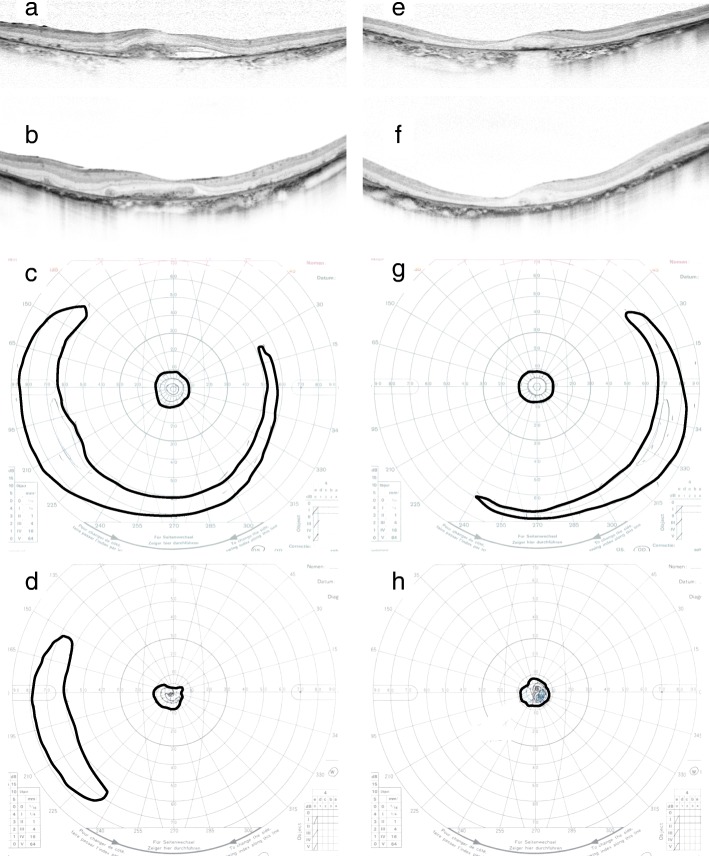
Optical coherence tomography and Goldmann perimetry data before and after 8 years of anti-VEGF therapy. Horizontal B-scan images of the left eye (**a**, **b**) and right eye (**e**, **f**) immediately before (**a**, **e**) and 8 years after (**b**, **f**) anti-VEGF therapy, respectively. Subfoveal choroidal neovascularization with serous retinal detachment was present at baseline (**a**). Exudative changes were well controlled and the fibrovascular membrane remained after 8 years of anti-VEGF therapy (**b**). Goldmann perimetry results for the left eye (**c**, **d**) and right eye (**g**, **h**) before (**c**, **g**) and 8 years after (**d**, **h**) anti-VEGF therapy, respectively. The bold lines represent V-4 isopters. The peripheral visual field was present before treatment in both eyes (**d**, **h**). However, after treatment, the peripheral visual field remained only in the left eye (**c**). VEGF, vascular endothelial growth factor

A 56-year-old woman who had autosomal dominant RP with a heterozygous *PRPH2* mutation (c.410G > A) complained of metamorphopsia in her left eye. Her best corrected visual acuity (BCVA) had declined from 1.0 (20/20) to 0.4 (20/50). Further examination revealed CNV with serous retinal detachment (Fig. 1). She was treated with as-needed injections for 2 years; however, she experienced a recurrence during which her vision deteriorated to 0.2 (20/100). Therefore, we switched to a bimonthly regimen that continued for 6 years. No recurrence was noted during that time, and her left visual acuity remained 0.2 (20/100). In total, the patient received 34 anti-VEGF injections in 8 years (bevacizumab × 2, pegaptanib × 2, ranibizumab × 11, aflibercept × 19, in that order).

**Fig. 2:**
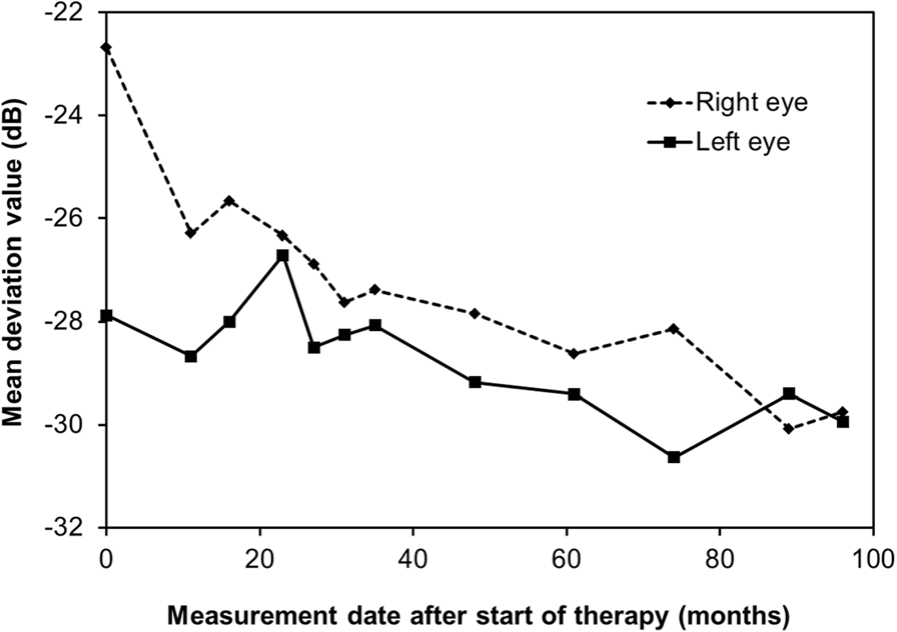
Change in the mean deviation value in both eyes after 8 years of treatment. The central visual field was assessed using the mean deviation (MD) value obtained using the Humphrey field analyser with the 10–2 SITA standard program. The MD values declined similarly in both eyes. The slope of the MD during the 8 years of treatment was − 0.68 dB/year and − 0.32 dB/year in the right eye and left eye, respectively. The results from the first examination for the right eye appear to be an outlier

The patient’s central visual field was assessed using the mean deviation (MD) value on a Humphrey field analyser with a 10–2 SITA standard program (Carl Zeiss Meditec, Inc., Dublin, CA). The MD values decreased similarly in both eyes (Fig. 2). The slope of the MD values during the 8-year treatment period was − 0.68 dB/year in the right eye (without CNV) and − 0.32 dB/year in the left eye (with CNV). Although her peripheral visual field loss was noted to have progressed based on Goldmann perimetry tests, her visual field in the left eye was preserved even after 8 years (Fig. 1). No serious adverse events were observed during treatment.

## Discussion

A previous case report demonstrated the effectiveness of a single injection of anti-VEGF (bevacizumab) for CNV cases associated with sectoral RP [7]. However, there was no information on the long-term outcome of anti-VEGF therapy due to the short 1-year follow-up period. In our patient, continuous injections of anti-VEGF over 8 years did not induce obvious progression of RP.

Although there was a decrease in BCVA due to CNV in this patient, no differences were detected in visual field loss between the two eyes. Goldmann perimetry test results showed similar progression of her peripheral visual field loss, and the MD slope was similar. Given that the values obtained for the right eye at the first examination may be an outlier, the progression of sensitivity loss of would be almost identical in both eyes and comparable with the reported average of − 0.46 dB/year [8]. Overall, long-term anti-VEGF therapy did not induce rapid progression of central or peripheral visual field loss in this patient.

This report has some limitations. First, it is based on a single case. We were not able to include other cases in this report because, although our institutional database includes approximately 1,000 patients with RP, only the one patient reported here received long-term anti-VEGF therapy. Furthermore, there are no similar reports in the literature, so it would be difficult to perform a case-series study. Second, the MD assessment may not have been adequate to estimate the progression of visual field loss in our patient because the value was low at baseline and could have been affected by the activity of CNV. However, we estimated the progression of visual field loss using Goldmann perimetry tests and a Humphrey field analyser with a 10–2 SITA standard program and obtained similar results.

## Conclusions

Continuous anti-VEGF therapy for 8 years for one eye in our patient with RP showed no negative effects, especially concerning the progression of visual field loss in comparison with the fellow eye. The outcome in our case suggests that long-term administration of an anti-VEGF agent for CNV and CME in patients with RP is likely to be safe, and hence, clinicians can consider this treatment option.

## Abbreviations

BCVA: best corrected visual acuity
CME: cystoid macular oedema
CNV: choroidal neovascularization
MD: mean deviation
RP: retinitis pigmentosa
VEGF: vascular endothelial growth factor

## References

[1] Hartong DT, Berson EL, Dryja TP (2006). Retinitis pigmentosa. Lancet.

[2] Marano F, Deutman AF, Leys A, Aandekerk AL (2000). Hereditary retinal dystrophies and choroidal neovascularization. Graefes Arch Clin Exp Ophthalmol.

[3] Rosenstein JM, Krum JM, Ruhrberg C (2010). VEGF in the nervous system. Organogenesis.

[4] Kurihara T, Westenskow PD, Bravo S, Aguilar E, Friedlander M (2012). Targeted deletion of Vegfa in adult mice induces vision loss. J Clin Invest.

[5] Yuzbasioglu E, Artunay O, Rasier R, Sengul A, Bahcecioglu H (2009). Intravitreal bevacizumab (Avastin) injection in retinitis pigmentosa. Curr Eye Res.

[6] Moustafa GA, Moschos MM (2015). Intravitreal aflibercept (Eylea) injection for cystoid macular edema secondary to retinitis pigmentosa - a first case report and short review of the literature. BMC Ophthalmol.

[7] Malik A, Sood S, Narang S (2010). Successful treatment of choroidal neovascular membrane in retinitis pigmentosa with intravitreal bevacizumab. Int Ophthalmol.

[8] Sayo A, Ueno S, Kominami T, Nishida K, Inooka D, Nakanishi A, Yasuda S, Okado S, Takahashi K, Matsui S (2017). Longitudinal study of visual field changes determined by Humphrey field analyzer 10-2 in patients with retinitis Pigmentosa. Sci Rep.

